# Multimodal imaging of photoreceptor and vascular changes in lamellar macular hole and epiretinal membrane-associated foveoschisis

**DOI:** 10.1007/s00417-026-07215-2

**Published:** 2026-03-28

**Authors:** Friedrich Caroli, Nikolaus Feucht, Mathias Maier, Nathalie Bleidißel

**Affiliations:** 1 Department of Ophthalmology, TUM University Hospital, School of Medicine and Health, Technical University of Munich, Ismaninger Str. 22, München, 81675 Germany; 2 Smile Eyes Airport Eyeclinic, Munich, Germany

**Keywords:** Lamellar macular hole, Epiretinal membrane–foveoschisis, Adaptive optics imaging, Optical coherence tomography angiography, Retinal microvasculature, Cone density mapping, Photoreceptor integrity

## Abstract

**Purpose:**

Lamellar macular hole (LMH) and epiretinal membrane–foveoschisis (ERM-FS) are distinct subtypes of lamellar macular defects, but clinical differentiation remains challenging. Adaptive optics (AO) and optical coherence tomography angiography (OCTA) may offer novel biomarkers for structural and vascular differences. This study aimed to evaluate photoreceptor integrity and macular microvasculature in LMH and ERM-FS compared with healthy controls.

**Design and participants:**

This prospective observational cross-sectional study was conducted at the Department of Ophthalmology, Technical University of Munich, between January 2022 and January 2025. Sixty-one eyes were included: 20 LMH, 21 ERM-FS, and 20 healthy controls. Exclusion criteria were other retinal diseases, high myopia, or prior intraocular surgery except cataract extraction.

**Methods:**

Best-corrected visual acuity (BCVA), spectral-domain OCT (SD-OCT), OCTA, and AO retinal imaging (rtx1, Imagine Eyes, Orsay, France) were performed.

**Main outcome measures:**

AO-derived metrics included cone density (CD), spacing, regularity, and dispersion. OCTA evaluated foveal and parafoveal vessel density (VD) in the superficial (SCP) and deep capillary plexus (DCP), foveal avascular zone (FAZ), and choriocapillaris flow. SD-OCT assessed central subfield thickness (CST), central subfield volume (CSV), and outer retinal integrity.

**Results:**

Mean [SD] age was 75.7 [7.5] years for LMH, 70.6 [8.7] for ERM-FS, and 71.5 [7.1] for controls. ERM-FS eyes showed increased cone spacing (e.g. at 4° inferior ERM-FS vs. LMH, *P* = 0.0159, Table 2) and dispersion (e.g. at 4° inferior ERM-FS vs. CG, *P* = 0.0115, Table 2) compared with LMH and controls, whereas cone density (e.g. at 0° ERM-FS vs. LMH, *P* =. 0.036, Table 2) and regularity (e.g. at 4° inferior ERM-FS vs. LMH, *P* = 0.060, Table 2) followed the same direction but did not remain statistically significant after correction for multiple testing. FAZ was larger in LMH than ERM-FS (0.342 vs. 0.236 mm²; *P* = 0.01). Foveal SCP VD was higher in ERM-FS than LMH and controls (28.16% vs. 22.53% vs. 21.82%; *P* = 0.01 ERM-FS vs. LMH). LMH eyes more frequently showed ellipsoid zone and external limiting membrane disruptions. ERM-FS was associated with greater CST and CSV.

**Conclusions:**

AO and OCTA reveal distinct photoreceptor and vascular alterations in LMH and ERM-FS. LMH is characterized by larger FAZ and more outer retinal disruption, whereas ERM-FS shows increased retinal thickness and more pronounced photoreceptor mosaic disorganization (increased cone spacing/dispersion), with trends toward lower cone density and reduced regularity. Higher foveal SCP VD in ERM-FS may, at least in part, reflect traction-related mechanical effects. Multimodal imaging may enhance diagnostic accuracy and monitoring in lamellar macular defects.

## Key messages

*****What is known***:**

- Lamellar macular hole (LMH) and epiretinal membrane–foveoschisis (ERM-FS) represent distinct entities within lamellar macular defects. Differences in photoreceptor integrity and retinal microvasculature are thought to underlie their distinct morphology and clinical course.

*****What is new***:**

- AO imaging demonstrates increased cone spacing and dispersion in ERM-FS compared with LMH and controls, with a consistent trend toward lower cone density and reduced cone regularity.
- OCTA reveals an enlarged FAZ in LMH and higher foveal superficial capillary plexus vessel density in ERM-FS, highlighting distinct tractional compared to degenerative features.

## Introduction

Lamellar macular defects affect the central retina and are associated with reduced visual acuity and metamorphopsia, significantly impacting quality of life [1]. The lamellar macular hole (LMH) was first described by Gass in the 1970s and initially used to distinguish full-thickness macular holes (FTMH) from macular pseudoholes (MPH) [2–6]. With the advent of high-resolution retinal imaging, particularly spectral-domain optical coherence tomography (SD-OCT), it became evident that lamellar macular defects are more heterogeneous than previously assumed based on clinical biomicroscopy.

In 2020, a consensus panel proposed an SD-OCT–based classification system distinguishing LMH, MPH, and epiretinal membrane–foveoschisis (ERM-FS) [7]. According to this definition, LMH is characterized by an irregular foveal contour, a foveal cavity with undermined edges, and signs of tissue loss, whereas ERM-FS features a contractile epiretinal membrane (ERM) with schisis at the Henle fiber layer level.

Adaptive optics (AO), originally developed in astronomy, has recently been translated into retinal imaging [8–11]. By minimizing ocular aberrations, AO enables high-resolution, in vivo visualization of cone photoreceptors, capillary networks, and retinal pigment epithelium at the cellular level. The rtx1 camera (Imagine Eyes, Orsay, France) was the first commercially available AO device for clinical ophthalmology. It has previously been used to quantify photoreceptor integrity in retinal diseases such as diabetic retinopathy, age-related macular degeneration, and glaucoma [12–18].

The aim of this study was to investigate photoreceptor morphology and retinal microvasculature in eyes with LMH and ERM-FS, using a multimodal imaging approach combining AO, SD-OCT, and OCT angiography (OCTA). OCTA provides noninvasive visualization of the superficial and deep capillary plexuses of the macula [19, 20]. This is, to our knowledge, the first study to apply AO imaging in LMH and ERM-FS and to quantify cone density, spacing, regularity, and dispersion, alongside vessel density (VD), foveal avascular zone (FAZ) area, and SD-OCT morphology in both conditions compared with a healthy control group.

## Methods

This prospective cross-sectional, single-center observational study was conducted in accordance with the tenets of the Declaration of Helsinki and was approved by the Ethics Committee of the Technical University of Munich (EK no.: 611/21 S-SR). Written informed consent was obtained from all participants prior to enrollment. The study followed the STROBE reporting guideline for observational studies.

### Participants

Participants were recruited at the Department of Ophthalmology, TUM University Hospital, between January 2022 and January 2025. Eligible patients had a diagnosis of either lamellar macular hole (LMH) or epiretinal membrane–foveoschisis (ERM-FS) confirmed by spectral-domain optical coherence tomography (SD-OCT). Diagnostic classification was based on the SD-OCT consensus definitions proposed in 2020 [7]. According to these criteria, LMH was defined by the presence of an irregular foveal contour, a foveal cavity with undermined edges, evidence of tissue loss, and intact outer retinal layers, in the absence of a full-thickness macular hole. In contrast, ERM-FS was defined by the presence of a contractile epiretinal membrane associated with schisis-like splitting at the level of the Henle fiber layer, retinal thickening, and tractional deformation without evidence of foveal tissue loss [7]. LMH and ERM-FS were considered mutually exclusive entities, as the presence of tissue loss is a defining feature of LMH, whereas ERM-FS is characterized by traction-induced retinal splitting without tissue loss. Eyes exhibiting overlapping features were carefully reviewed; cases not allowing a clear classification were excluded to ensure diagnostic consistency.

Healthy individuals without retinal pathology were included as controls; the control group was selected to ensure comparable mean age across all three study groups. Healthy individuals without retinal pathology were included as controls. The control group was selected to ensure comparable mean age across all three study groups. Exclusion criteria comprised secondary LMH or ERM-FS (e.g., due to high myopia or uveitis), glaucoma, neovascular age-related macular degeneration, pathologic myopia, and history of intraocular surgery other than cataract extraction.

### Imaging and assessments

All participants underwent a standardized ophthalmologic examination, including best-corrected visual acuity (BCVA) testing, intraocular pressure measurement, slitlamp biomicroscopy, indirect ophthalmoscopy, and Amsler grid assessment for metamorphopsia. SD-OCT imaging was performed using a SPECTRALIS OCT device (Heidelberg Engineering, Heidelberg, Germany). For diagnostic classification, high-resolution macular star scan protocols centered on the fovea were used, consisting of 48 radial B-scans with an angular spacing of 3.75°, enabling detailed assessment of foveal architecture and tractional changes. In addition, horizontal volume scans comprising 145 B-scans with an inter-scan distance of approximately 30 μm were acquired for quantitative measurements, including central subfield thickness (CST), central subfield volume (CSV), total macular volume, and evaluation of the ellipsoid zone (EZ) and external limiting membrane (ELM).

OCTA imaging (Optovue RTVue XR Avanti, AngioVue software) was performed to evaluate macular vascular parameters. Foveal and parafoveal vessel density (VD) in the superficial capillary plexus (SCP) and deep capillary plexus (DCP) were assessed using 6 × 6-mm scans. The foveal avascular zone (FAZ) and choriocapillaris flow area were measured using 3 × 3-mm scans. Manual measurement of subfoveal choroidal thickness (SCT) was performed on SD-OCT images using the 1:1 μm setting from Bruch’s membrane to the sclerochoroidal junction.

**Fig. 1:**
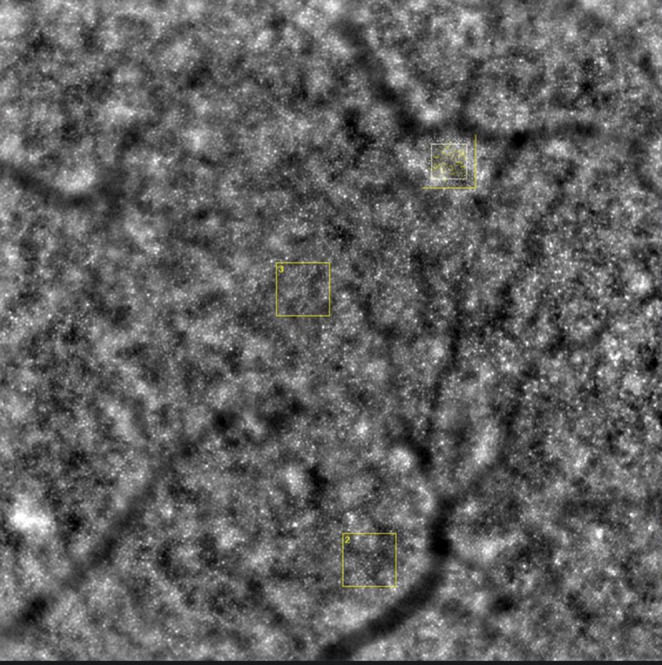
Image of the acquired 2° nasal area of an eye with a Lamellar Macular Hole. *The three yellow rectangles represent the regions of interest in which the evaluation of the Cone density*,* Cone spacing and Cone dispersion was carried out in this area*

**Fig. 2:**
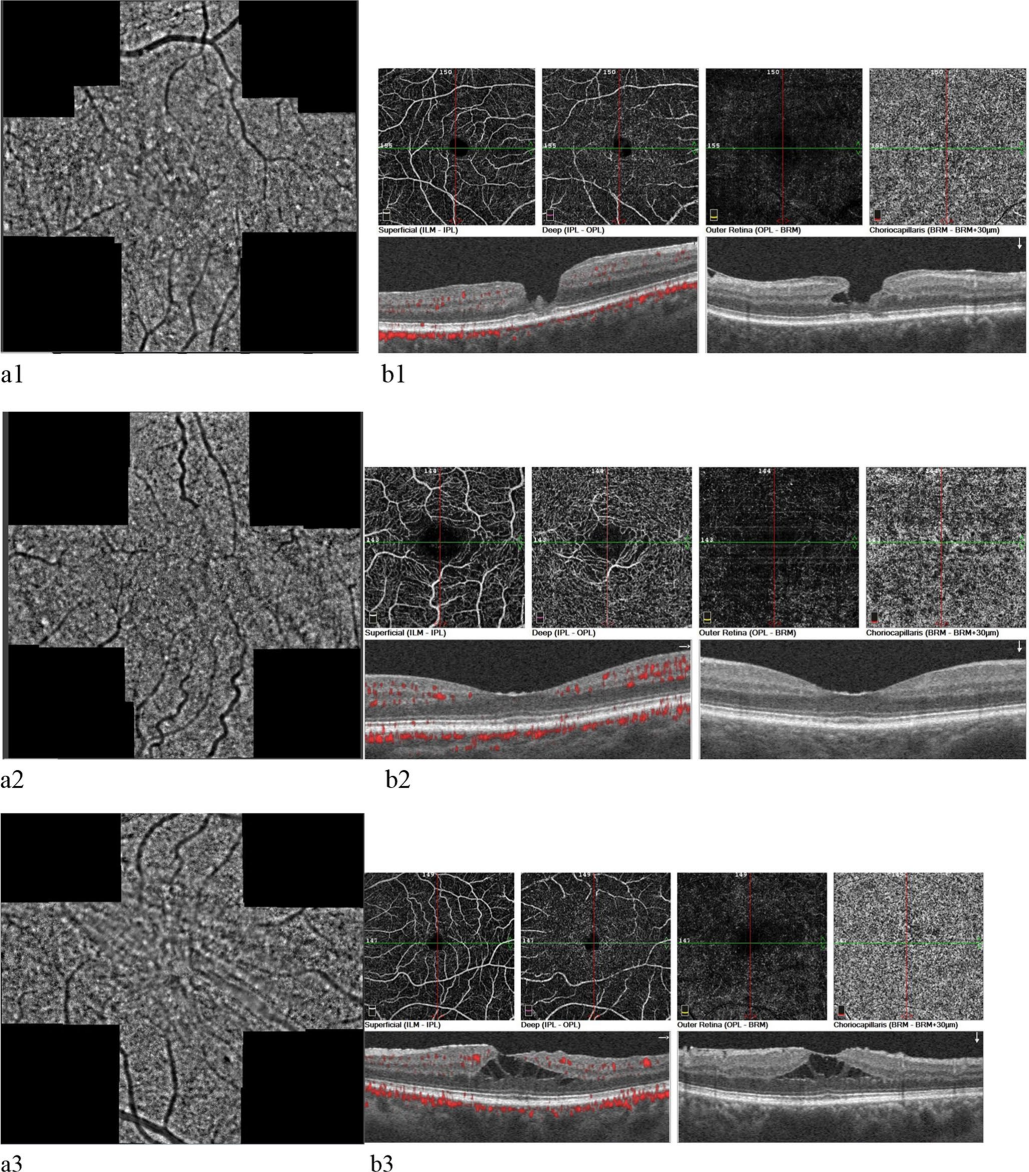
Multimodal imaging examples from the three study groups: (1) lamellar macular hole (LMH), (2) epiretinal membrane–foveoschisis (ERM-FS), and (3) healthy control. For each group, rtx1 montages (a1–a3) composed of nine acquired aerial images (0°, 2° temporal, superior, nasal, inferior; and 4° temporal, superior, nasal, inferior) are shown alongside corresponding optical coherence tomography angiography images (b1–b3). Panels a1/b1 represent the LMH group, a2/b2 the ERM-FS group, and a3/b3 the control group. *(a1) rtx1 Montage of the 9 acquired aerials ( 0°*,* 2° temporal*,* 2° superior*,* 2° nasal*,* 2° inferior*,* 4° temporal*,* 4° superior*,* 4° nasal*,* 4° inferior)*. *(b1) Optical coherence tomography angiography imaging 6 × 6-mm scan*. *(a2) rtx1 Montage of the 9 acquired aerials ( 0°*,* 2° temporal*,* 2° superior*,* 2° nasal*,* 2° inferior*,* 4° temporal*,* 4° superior*,* 4° nasal*,* 4° inferior)*. *(b2) Optical coherence tomography angiography imaging 3 × 3-mm scan*. *(a3) rtx1 Montage of the 9 acquired aerials ( 0°*,* 2° temporal*,* 2° superior*,* 2° nasal*,* 2° inferior*,* 4° temporal*,* 4° superior*,* 4° nasal*,* 4° inferior)*. *(b3) Optical coherence tomography angiography imaging 6 × 6-mm scan*

High-resolution photoreceptor imaging was obtained with the rtx1 adaptive optics camera (Imagine Eyes, Orsay, France). Nine retinal locations per eye were imaged: one at the foveal center (0°) and eight parafoveal locations at 2° and 4° eccentricity along the nasal, temporal, superior, and inferior meridians. Photoreceptor analysis was conducted using AOdetect software (Imagine Eyes, Orsay, France). In each location, three regions of interest (ROIs) were manually selected, excluding vascular structures, artifacts, or areas lacking a visible cone mosaic (Fig. 1). Quantitative AO metrics included cone density (cells/mm²), cone spacing (µm, center-to-center distance), cone regularity (percentage of cones with 5–7 neighbors), and cone dispersion (coefficient of variation in spacing). If fewer than three suitable ROIs were present in a location, that region was excluded from analysis. Pupillary dilation was performed with mydriatics prior to AO imaging. Representative OCTA and RTx images of LMH, ERM-FS, and control eyes are shown in Fig. 2.

### Statistical analysis

All statistical analyses were performed using R version 4.2.3 (R Foundation for Statistical Computing). Decimal BCVA was converted to logarithm of the minimum angle of resolution (LogMAR). Depending on data distribution and variable type, global comparisons across the three groups (CG, MSF, and ERM-FS) were conducted using one-way analysis of variance (ANOVA, †), the Kruskal-Wallis test (‡), the Pearson’s chi-squared test (#), or Fisher’s exact test (§). P Values less than 0.05 were considered statistically significant and are highlighted in bold in the results. For variables with a significant P Value, post-hoc pairwise comparisons were performed. Post hoc comparisons were Bonferroni-adjusted. To account for multiple testing, a Bonferroni correction was applied, and a P value less than 0.0167 was considered statistically significant for all post-hoc analyses.

Sample size was determined in consultation with biometric experts at the TUM Institute for AI and Computer Science. Given the novelty of AO imaging in LMH and ERM-FS and limited prior data on OCTA metrics in lamellar macular defects, 20 eyes per group were deemed appropriate for exploratory analysis.

## Results

Sixty-one eyes of 60 participants were included in the study: 20 eyes with LMH, 21 eyes with ERM-FS, and 20 eyes from healthy controls (CG). The mean [SD] age was 75.7 [7.5] years in the LMH group, 70.6 [8.7] years in the ERM-FS group, and 71.5 [7.1] years in the CG group. No significant age difference was observed between groups (Table 1). The proportion of female participants was 70% in the LMH group, 80% in the ERM-FS group, and 45% in the CG.

**Table 1:** Demographics and Optical Coherence Tomography (OCT) measurements

Measures	LMH (+-SD / %)	ERMFS (+-SD / %)	CG (+-SD / %)	*P* Value	*P* Value 1	95% CI (1)	*P* Value 2	95% CI (2)	*P* Value 3	95% CI (3)
Age	75.7 +- 7.52	70.6 +- 8.73	71.5 +- 7.06	0.09 ‡						
BCVA (LogMAR)	0.263 +- 0.246	0.178 +- 0.167	0.0648+- 0.064	**< 0.01** **†**	0.242	-0.155 to 0.058	**< 0.01**	-0.222 to − 0.058	0.045	-0.204 to 0.00009
BCVA (decimal)	0.613+-0.243	0.710+-0.247	0.87+-0.122	**< 0.01** **†**	0.25	-0.10 to 0.27	**< 0.01**	0.10 to 0.40	0.045	-0.30 to 0.00
Positive Amsler test	12 (60)	13 (61.90)	0 (0)	**< 0.01 #**	1.0	-0.32 to 0.28	**< 0.01**	0.39 to 0.81	**< 0.01**	0.41 to 0.83
Sex				0.05 #						
Male, n (%)	6(30)	4 (20)	11 (55)							
Female, n (%)	14 (70)	16 (80)	9 (45)							
Lens status				0,8 #						
Phak, n (%)	9 (45)	11 (52.4)	11 (55)							
Pseudophak, n (%)	11 (55)	10 (47.6)	9 (45)							
EZ disruption, n (%)	12 (60)	5 (25)	0 (0)	**< 0.01 #**	0.03	-0.64 to − 0.06	**< 0.01**	-0.81 to − 0.39	0.0168	0.06 to 0.44
ELM disruption, n (%)	7 (35)	4 (20)	0 (0)	**0.01 §**	0.5	0.08 to 2.4	< 0.01	-0.56 to − 0.14	0.1	0.02 to 0.38
ERM, n (%)	8 (40)	20 (100)	0 (0)	**< 0.01 #**	**< 0.01**	5.3 to Inf	**< 0.01**	0.0 to 0.4	**< 0.01**	51.7 to Inf
EPR, n (%)	6 (30)	21 (100)	0 (0)	**< 0.01 #**	**< 0.01**	0.0 to 0.7	**< 0.01**	0.0 to 0.7	-	-
Central subfield thickness in µm	298.0 +- 41.2	410.0 +- 70.4	279.0 +- 22.4	**< 0.01 ‡**	**< 0.01**	75.50 to 149.57	0.46	-56.08 to 18.88	**< 0.01**	94.10 to 168.17
Central subfield volume in mm³	0.234 +- 0.03	0.322 +- 0.05	0.22 +- 0.01	**< 0.01 ‡**	**< 0.01**	0.059 to 0.118	0.5	-0.044 to 0.016	**< 0.01**	0.073 to 0.132
Total macular volume in mm³	8.76 +- 0,61	9.15 +- 1.60	8.32 +- 0,51	0.05 ‡						
Subfoveal choroidal thicknessin µm	211 +- 72.4	207 +-56.2	217 +- 46.2	0.08 ‡						

*BCVA* Best-corrected visual acuity, *LogMAR* Logarithm of the minimum angle of resolution, *SD* Standard deviation, *EZ* Ellipsoid zone, *ELM* External limiting membrane, *ERM* Epiretinal membrane, *EPR* Epiretinal proliferation, *95*% *CI* 95% confidence interval

P Value was determined for the three-group comparison (CG, MSF, and ERMFS) using: † = ANOVA; ‡ = Kruskal-Wallis test; # = Pearson’s chi-squared test; § = Fisher’s exact test. Significant P values (*P* < 0.05) are highlighted in bold. For variables with a significant P Value, post-hoc pairwise comparisons (P value 1 = comparison between LMH and ERMFS; P value 2 = comparison between LMH and CG; P value 3 = comparison between ERMFS and CG) were performed. To account for multiple testing, a Bonferroni correction was applied, with statistical significance for post-hoc tests set at *P* < 0.0167

Baseline visual acuity was significantly worse in both disease groups compared to the CG. Mean [SD] LogMAR BCVA was 0.26 [0.25] in LMH, 0.18 [0.17] in ERM-FS, and 0.06 [0.06] in controls (*P* < 0.05 for both comparisons vs. CG). BCVA did not differ significantly between the LMH and ERM-FS groups (*P* = 0.242).

### Adaptive optics imaging

Eyes with ERM-FS demonstrated a trend toward lower cone density (CD) than those with LMH across all regions (e.g., 0°: 16,218 vs. 19,637 photoreceptors/mm², *P* = 0.036, Table 2). Although the overall ANOVA indicated a significant group effect, post hoc pairwise comparisons did not remain statistically significant after Bonferroni correction.

**Table 2:** Comparison of the cone mosaic in eyes with Lamellar Macular Hole, Epiretinal Membrane-Foveoschisis and the healthy control group

Measures	Eccentricity	LMH (+-SD)	ERMFS (+-SD)	CG (+-SD)	*P* Value	*P* Value 1	95% CI (1)	*P* Value 2	95% CI (2)	*P* Value 3	95% CI (3)
Cone density (photoreceptors/mm2)	0°	19,637 +- 4227	16,218 +- 1477	21,660 +- 3432	**0.01 †**	0.036	-7050 to -618,67	0.53	-1815 to 5141	**0.001**	-8905 to -1875
Cone density (photoreceptors/mm2)	2° Temporal	18,390 +- 3876	16,841 +- 2990	22,561 +- 3627	**0.007 †**	0.17	-5598 to 2412	0.02	675 to 7882	**0.004**	-9045 to -1894
Cone density (photoreceptors/mm2)	2° Superior	18,290 +- 3316	17,389 +- 1988	21,339 +- 2300	**0.015** ‡	0.51	-3802 to 2000	0.02	491 to 5607	**0.007**	-9045 to -1894
Cone density (photoreceptors/mm2)	2° Nasal	19,103 +- 2538	17,567 +- 2283	21,900 +- 2799	**0.008 ‡**	0.22	-4140 to 1068	0.02	387 to 5206	**0.005**	-7121 to -1544
Cone density (photoreceptors/mm2)	2° Inferior	19179.90 +- 3349.81	16151.48 +- 3131.35	22417.97 +- 4524.22	**0.008 ‡**	0.06	-6274 to 217	0.08	-377 to 6853	**0.004**	-10,224 to -2308
Cone density (photoreceptors/mm2)	4 ° Temporal	15675.91 +- 3349.81	16624.71 +- 3131.35	19484.50 +- 4264.24	0.10 ‡						
Cone density (photoreceptors/mm2)	4° Superior	15,134 +- 3223	13 860 +- 2469	16,389 +- 2690	0.24 ‡						
Cone density (photoreceptors/mm2)	4° Nasal	17,528 +- 2992	15,552 +- 2533	19,425 +- 3036	**0.02 ‡**	0.11	-4432 to 481	0.17	-907 to 4701	**0.009**	-6613 to -1131
Cone density (photoreceptors/mm2)	4 ° Inferior	16,753 +- 4065	12,667 +- 2703	19,814 +- 3078	**0.0002 ‡**	0.02	-7490 to − 680	0.09	-563 to 6685	**< 0.001**	-9922 to -4370
Cone Spacing in µm	0°	8.0 +- 0.96	8.63 +- 0.351	7.59 +- 0.601	**0.01 ‡**	0.045	0.11 to 1.36	0.5	-1.10 to 0.40	**< 0.001**	0.46 to 1.66
Cone Spacing in µm	2° Temporal	8.2 +- 0.84	8.55 +- 0.793	7.40 +- 0.588	**0.008 ‡**	0.16	-0.43 to 1.06	0.03	-1.53 to − 0.18	**0.004**	0.44 to 1.79
Cone Spacing in µm	2° Superior	8.18 +- 0.67	8.36 +- 0.475	7.59 +- 0.401	**0.018 ‡**	0.66	-0.64 to 0.92	**0.004**	-1.14 to − 0.06	**0.009**	0.21 to 1.33
Cone Spacing in µm	2° Nasal	8.02 +- 0.549	8.34 +- 0.523	7.51 +- 0.476	**0.01 ‡**	0.21	-0.24 to 1.03	0.03	-0.97 to − 0.01	**0.0160**	0.30 to 1.46
Cone Spacing in µm	2° Inferior	8.04 +- 0.735	8.69 +- 0.700	7.50 +- 0.845	**0.01 ‡**	0.04	0.10 to 1.28	0.04	-1.11 to − 0.02	**0.025**	0.20 to 1.97
Cone Spacing in µm	4 ° Temporal	8.97 +- 1.32	8.59 +- 0.636	8.03 +- 0.885	0.16 ‡						
Cone Spacing in µm	4° Superior	8.98 +- 0.72	9.35 +- 0.742	8.63 +- 0.697	0.15 ‡						
Cone Spacing in µm	4° Nasal	8.93 +- 0.694	8.83 +- 0.621	7.98 +- 0.620	**0.03 ‡**	0.16	-0.28 to 1.04	0.31	-1.04 to 0.27	**0.018**	0.17 to 1.54
Cone Spacing in µm	4 ° Inferior	8.59 +- 0.896	9.86 +- 1.35	7.93 +- 0.707	**0.001 ‡**	**0.0159**	0.31 to 2.18	0.08	-1.41 to 0.23	**0.0005**	1.05 to 2.55
Cone Regularity in %	0°	89.6 +- 5.25	86.6 +- 3.59	92.2 +- 2.97	**0.04 ‡**	0.16	-7.31 to 1.29	0.17	-1.17 to 6.33	**0.006**	-9.20 to − 1.97
Cone Regularity in %	2° Temporal	89.8 +-2.75	88.3 +- 5.75	93.1 +- 3.09	0.06 ‡						
Cone Regularity in %	2° Superior	90.1 +- 2.76	90.5 +- 2.53	91.7 +- 1.75	0.28 ‡						
Cone Regularity in %	2° Nasal	90.3 +- 3.09	90.6 +- 3.09	92.5 +- 1.53	0.10 **†**						
Cone Regularity in %	2° Inferior	91.2 +- 3.27	90.4 +- 3.60	90.5 +- 3.75	0.85 ‡						
Cone Regularity in %	4 ° Temporal	89.4 +- 4.09	93.2 +- 3.55	91.2 +- 4.52	0.129 ‡						
Cone Regularity in %	4° Superior	88.7 +- 5.06	89.0 +- 2.61	88.4 +- 4.93	0.97 ‡						
Cone Regularity in %	4° Nasal	90.4 +- 3.41	90.3 +- 6.19	90.7 +- 3.84	0.98 ‡						
Cone Regularity in %	4 ° Inferior	91.0 +- 4.44	88.6 +- 4.18	92.3 +- 2.14	**0.03 †**	0.06	-5.26 to 0.46	0.54	-4.05 to 1.45	**0.0111**	-5.71 to − 1.69
Cone Dispersion in %	0°	16.3 +- 4.81	18.2 +- 1.82	13.7 +- 2.12	**0.02 †**	0.17	-2.65 to 5.80	0.31	-6.60 to 0.93	**0.0007**	2.38 to 6.73
Cone Dispersion in %	2° Temporal	16.4 +- 4.1	16.5 +- 3.68	12.7 +- 1.44	**0.03 †**	0.8	-3.92 to 3.40	-1.62	-7.68 to − 0.51	0.04	0.08 to 7.37
Cone Dispersion in %	2° Superior	16.3 +- 4.13	13.2 +- 2	13.9 +- 0.691	0.13 **†**						
Cone Dispersion in %	2° Nasal	15.4 +- 2.83	15.7 +- 2	14.3 +- 1.64	0.40 ‡						
Cone Dispersion in %	2° Inferior	15.7 +- 4.78	17.2 +- 4.9	14.2 +- 1.74	0.16 **†**						
Cone Dispersion in %	4 ° Temporal	18.1 +- 5.64	14.6 +- 4.2	14.0 +- 4.12	0.13 ‡						
Cone Dispersion in %	4° Superior	17.1 +- 3.41	17.2 +- 4.45	15.7 +- 3.82	0.66 ‡						
Cone Dispersion in %	4° Nasal	14.9 +- 3.99	17.0 +- 4.34	16.6 +- 8.3	0.3 **†**						
Cone Dispersion in %	4 ° Inferior	15.1 +- 3.83	19.2 +- 4.49	13.8 +- 2.75	**0.01 ‡**	0.8	-3.36 to 5.95	0.08	-8.66 to 0.48	**0.0115**	1.08 to 9.69

*95*% *CI* 95% confidence interval

P Value was determined for the three-group comparison (CG, MSF, and ERMFS) using: † = ANOVA; ‡ = Kruskal-Wallis test; # = Pearson’s chi-squared test; § = Fisher’s exact test. Significant P values (*P* < 0.05) are highlighted in bold. For variables with a significant P Value, post-hoc pairwise comparisons (P value 1 = comparison between LMH and ERMFS; P value 2 = comparison between LMH and CG; P value 3 = comparison between ERMFS and CG) were performed. To account for multiple testing, a Bonferroni correction was applied, with statistical significance for post-hoc tests set at *P* < 0.0167

Compared with the CG, the ERM-FS group had significantly reduced CD at the fovea and in all 2° parafoveal quadrants, as well as at 4° nasal and inferior. The LMH group showed a trend toward lower CD values compared with the CG; although the overall group effect was significant, post hoc pairwise comparisons did not remain statistically significant after correction for multiple testing (Table 2).

Cone spacing was highest in the ERM-FS group, followed by LMH, and lowest in the CG across all regions. Spacing was significantly greater in ERM-FS compared to LMH at 4° inferior. Compared to controls, ERM-FS showed significantly increased spacing in 7 of 9 regions; LMH showed significantly greater spacing than CG in 2° superior (Table 2).

Cone regularity was highest in the CG and lowest in ERM-FS. Significant differences in regularity were observed at 0° and 4° inferior between CG and ERM-FS (Table 2).

Cone dispersion followed a similar pattern: highest in ERM-FS, intermediate in LMH, and lowest in the CG. Compared to CG, ERM-FS had significantly increased dispersion at 0° and 4° inferior (Table 2).

### OCT angiography

Mean [SD] foveal avascular zone (FAZ) area was significantly larger in LMH (0.342 [0.175] mm²) than in ERM-FS (0.236 [0.090] mm²) and the CG (0.234 [0.100] mm²; *P* = 0.01). Foveal vessel density (VD) in the superficial capillary plexus (SCP) was significantly higher in ERM-FS (28.16% [7.86]) compared to LMH (22.53% [8.06]; *P* = 0.01) and controls (21.82% [6.70]; *P* = 0.008). No significant differences were found in parafoveal SCP VD across groups.

In the deep capillary plexus (DCP), the LMH group had the lowest foveal VD (30.27% [9.25]), significantly lower than the CG (37.27% [7.34]; *P* = 0.01). ERM-FS showed intermediate values (35.08% [9.32]) without statistically significant differences. Parafoveal VD in the DCP did not differ significantly between groups.

Choriocapillaris flow area was greatest in the CG (0.833 [0.332] mm²), followed by LMH (0.717 [0.392] mm²) and ERM-FS (0.653 [0.332] mm²); however, these differences were not statistically significant (Table 3).

**Table 3:** Optical Coherence Tomography Angiography (OCT - A) measurements

Measures	LMH Mean ± SD	ERMFS Mean ± SD	CG Mean ± SD	*P* Value	*P* Value 1	95% CI (1)	*P* Value 2	95% CI (2)	*P* Value 3	95% CI (3)
Foveal SCP VD in %	22.53 +- 8.06	28.16 +- 7.86	21.82 +- 6.70	**0**,**01 †**	**0.01**	-11.95 to -1.00	0.76	-5.45 to 4.04	**0.008**	-14.6 to -0.1
Parafoveal SCP VD in %	44.16 +- 7.38	42.08 +- 5.47	44.84 +- 5.85	0.36 ‡						
Foveal DCP VD in %	30.27 +- 9.25	35.08 +- 9.32	37.27 +- 7.34	**0**,**04** ‡	0.11	-1.13 to 10.8	**0.01**	1.66 to 12.3	0.41	-7.56 to 3.17
Parafoveal DCP VD in %	49.8 +- 4.48	48.3 +- 5.75	48.2 +- 4.80	0,52 ‡						
FAZ in mm²	0.342 +- 0.175	0.236 +- 0.09	0.234 +- 0.10	**0**,**01 †**	**0.01**	0.04 to 0.12	0.02	-0.2 to -0.01	0.82	-0.07 to 0.09
Choriokapillaris Flow in mm²	0.717 +- 0.392	0.653 +- 0.332	0.833 +- 0.332	0,26 ‡						

*95*% *CI* 95% confidence interval

P Value was determined for the three-group comparison (CG, MSF, and ERMFS) using: † = ANOVA; ‡ = Kruskal-Wallis test; # = Pearson’s chi-squared test; § = Fisher’s exact test. Significant P values (*P* < 0.05) are highlighted in bold. For variables with a significant P Value, post-hoc pairwise comparisons (P value 1 = comparison between LMH and ERMFS; P value 2 = comparison between LMH and CG; P value 3 = comparison between ERMFS and CG) were performed. To account for multiple testing, a Bonferroni correction was applied, with statistical significance for post-hoc tests set at *P* < 0.0167

## SD-OCT morphology

Outer retinal layer disruption was more common in the LMH group. EZ disruption was present in 60% of LMH eyes and 25% of ERM-FS eyes; ELM disruption occurred in 35% and 20% of eyes, respectively.

ERM was present in all ERM-FS eyes by definition and in 40% of LMH eyes. Epiretinal proliferation (ERP) was seen in 30% of LMH eyes and absent in the CG.

ERM-FS eyes demonstrated the highest central subfield thickness (CST), central subfield volume (CSV), and total macular volume, with significant differences compared to the CG in all three parameters. Compared to LMH, CST and CSV were also significantly higher in ERM-FS. While LMH eyes had higher values than controls, these differences were not statistically significant. Subfoveal choroidal thickness (SCT) was similar across all groups: 211 μm (LMH), 207 μm (ERM-FS), and 217 μm (CG), with no significant group differences. An overview of all OCT parameters is provided in Table 1.

## Discussion

This multimodal imaging study using adaptive optics (AO), optical coherence tomography (OCT), and OCT angiography (OCTA) revealed distinct structural and vascular differences between eyes with lamellar macular hole (LMH), epiretinal membrane-associated foveoschisis (ERM-FS), and healthy controls. To our knowledge, this is the first study to apply AO-based photoreceptor quantification in LMH and ERM-FS and to compare these subtypes using OCTA according to the updated SD-OCT-based classification [7].

Eyes with ERM-FS exhibited the most pronounced alterations of the photoreceptor mosaic, characterized by significantly increased cone spacing and dispersion, along with a consistent trend toward lower cone density and reduced cone regularity across several foveal and parafoveal regions. The LMH group generally showed intermediate values, while healthy controls demonstrated the most preserved photoreceptor structure. These findings suggest that photoreceptor mosaic disruption in ERM-FS is primarily reflected by spatial disorganization rather than absolute cone loss, potentially related to tractional forces and inner retinal distortion associated with epiretinal membranes. The observed trends toward lower cone density and regularity further support this interpretation but should be considered exploratory, as they did not remain statistically significant after correction for multiple testing.

Our study aligns with the pattern described in the literature in which lower cone density is associated with increased spacing, lower regularity, and higher dispersion [13, 15, 21]. In the present study, this pattern was most consistently reflected by significant changes in cone spacing and dispersion, while density and regularity changes followed the same direction but did not reach statistical significance after correction.

Our results are supporting previous AO imaging studies in full-thickness macular hole (FTMH), a more severe entity within the spectrum of macular defects. Oquendo et al. demonstrated lower parafoveal cone density and increased spacing in eyes with FTMH, paralleling the structural abnormalities observed in our ERM-FS group [22].

FTMH studies support the theory that neuronal and retinal elements are affected during disease development, resulting in parafoveal photoreceptor mosaic impairment [23, 24]. Although direct comparisons are limited due to differences in disease severity, age, and analytic methods, our findings support the hypothesis that structural integrity of the photoreceptor mosaic is compromised beyond the immediate foveal center in lamellar macular defects. The more pronounced abnormalities in ERM-FS compared to LMH may reflect the additional structural stress from the associated membrane traction.

OCTA findings further differentiated LMH and ERM-FS. Eyes with LMH had a significantly enlarged foveal avascular zone (FAZ) compared to both ERM-FS and controls, in line with prior studies suggesting capillary rarefaction and retinal remodeling in degenerative LMH [25–27]. In contrast, ERM-FS eyes showed higher foveal vessel density (VD) in the superficial capillary plexus (SCP) compared to controls. This finding may reflect traction-related microvascular remodeling and vascular crowding caused by tangential forces exerted by the epiretinal membrane, which can mechanically displace perifoveal capillaries toward the foveal center rather than reflect true pathological microvascular remodeling. As FAZ size directly influences SCP VD measurements, a reduced FAZ may inherently result in higher calculated vessel density values [28–31]. In addition, compensatory capillary dilation in response to altered retinal biomechanics and increased metabolic demand of the distorted inner retina may contribute to the observed increase in SCP VD. Furthermore, segmentation-related influences inherent to OCTA analysis in the presence of retinal traction and layer distortion cannot be fully excluded and may partially affect quantitative SCP measurements in ERM-FS.

Moreover, axial length related magnification effects may alter the effective retinal area captured by OCTA, which should be considered when interpreting quantitative OCTA measurements.

The absence of significant differences in the parafoveal SCP and deep capillary plexus (DCP) VD suggests that vascular alterations in ERM-FS are predominantly confined to the foveal region rather than reflecting a diffuse microvascular involvement. These OCTA findings should be interpreted as structural-functional imaging correlates influenced by mechanical traction, rather than as definitive evidence of intrinsic vascular remodeling. Further prospective studies with advanced segmentation correction are warranted. However, these findings are in agreement with earlier reports indicating increased SCP perfusion in eyes with ERM, potentially due to inner retinal distortion and glial proliferation [32, 33].

Subfoveal choroidal thickness did not differ significantly between groups, mirroring inconsistent findings in the literature [31, 34–36]. However, larger studies are warranted to clarify potential choroidal involvement in lamellar macular pathology.

On SD-OCT, eyes with ERM-FS exhibited significantly greater central subfield thickness and macular volume than those with LMH or controls, consistent with the tractional nature of the disease [37, 38]. Conversely, LMH was more frequently associated with outer retinal layer disruption, including ellipsoid zone (EZ) and external limiting membrane (ELM) breaks - findings aligned with the degenerative nature of LMH and its impact on photoreceptor integrity [39].

Limitations of the study include the modest sample size and cross-sectional design, which limit conclusions regarding disease progression and causality. Furthermore, although segmentation and quality control were meticulously performed, some areas of poor image quality or undetectable cone mosaic required exclusion, potentially biasing measurements in advanced disease. Regions of interest were defined according to a standardized foveal and parafoveal scan protocol to ensure intergroup comparability; however, targeted AO analysis of OCT-defined outer retinal disruption areas may provide additional insights in future longitudinal studies. In addition, AO findings should be interpreted cautiously. At present, AO-derived photoreceptor metrics primarily provide high-resolution microstructural information and are best regarded as exploratory imaging biomarkers rather than direct clinical outcome measures. Further longitudinal studies are required to clarify their prognostic value and functional relevance in LMH and ERM-FS.

## Conclusions

In this study, AO-based imaging revealed structural alterations of the photoreceptor mosaic in ERM-FS and LMH, with ERM-FS showing the most pronounced disruption. OCTA analysis demonstrated that FAZ enlargement is a distinguishing feature of LMH, while elevated foveal VD in the SCP characterizes ERM-FS, likely reflecting traction-related mechanical effects rather than intrinsic vascular pathology. These structural and vascular differences underscore the pathophysiologic divergence between tractional and degenerative subtypes of lamellar macular defects.

Our findings suggest that AO imaging and OCTA provide complementary insights into disease mechanisms and may support the development of imaging biomarkers for disease classification and progression. In future research, longitudinal studies with larger cohorts and multimodal imaging at multiple time points are needed to establish prognostic markers and evaluate the potential role of AO and OCTA in guiding surgical decision-making.

## Data Availability

The data that support the findings of this study are available from the corresponding author, upon reasonable request.
